# Sestrin2 Suppression Promotes Endothelial–Mesenchymal Transition and Exacerbates Methylglyoxal-Induced Endothelial Dysfunction

**DOI:** 10.3390/ijms252413463

**Published:** 2024-12-16

**Authors:** Shahenda Salah Abdelsalam, Muhammad Ammar Zahid, Sarah Khalaf Ghanem, Abbas Khan, Aijaz Parray, Abdelali Agouni

**Affiliations:** 1 Department of Pharmaceutical Sciences, College of Pharmacy, QU Health, Qatar University, Doha P.O. Box 2713, Qatar; sa1512854@qu.edu.qa (S.S.A.); mz1912625@qu.edu.qa (M.A.Z.); sg1301626@qu.edu.qa (S.K.G.); 2 Department of Biological Sciences, School of Medical and Life Sciences (SMLS), Sunway University, Bandar Sunway 47500, Malaysia; abbask@sunway.edu.my; 3 The Neuroscience Institute, Academic Health System, Hamad Medical Corporation, Doha P.O. Box 3050, Qatar; aparray@hamad.qa

**Keywords:** Sestrin2, methylglyoxal, endothelial dysfunction, endothelial–mesenchymal transition

## Abstract

Sestrin2 (SESN2) is a stress-inducible protein known for its cytoprotective functions, but its role in diabetic vascular complications remains unclear. This study investigated the impact of SESN2 on methylglyoxal (MGO)-induced endothelial–mesenchymal transition (EndMT). Human endothelial cells were transfected with SESN2 siRNA duplexes to silence SESN2 expression, followed by MGO treatment. SESN2 knockdown significantly exacerbated MGO-induced oxidative stress, as evidenced by the reduced expression of antioxidant markers. Furthermore, SESN2 silencing enhanced the inflammatory response to MGO, demonstrated by the increased levels of pro-inflammatory cytokines. Notably, SESN2 deficiency promoted EndMT, a key process in diabetes-induced cardiovascular complications, as shown by the increased expression of mesenchymal markers and the decreased expression of endothelial markers. These findings suggest that SESN2 plays a critical protective role in endothelial cells against MGO-induced damage. The study provides novel insights into the molecular mechanisms underlying diabetic cardiovascular complications and identifies SESN2 as a potential therapeutic target for preventing endothelial dysfunction in diabetes. Our results indicate that SESN2 downregulation may contribute to the pathogenesis of diabetic vascular complications by promoting EndMT, increased oxidative stress, and inflammation.

## 1. Introduction

Endothelial dysfunction is a critical event in the pathogenesis of various cardiovascular disorders, including hypertension and atherosclerosis. The endothelium plays a key role in maintaining vascular homeostasis by controlling vascular tone, permeability, and immune responses [1]. Under pathological insults, such as oxidative stress, inflammation, and metabolic dysregulation, endothelial cells (ECs) lose their protective properties and undergo structural and functional changes [2]. One such change is an endothelial-to-mesenchymal transition (EndMT), a dynamic process in which endothelial cells undergo a phenotypic transformation, losing their characteristic endothelial features and acquiring a mesenchymal-like phenotype. During this process, the endothelial cells lose their typical cell–cell junctions and gain increased migratory and invasive abilities, as well as the ability to secrete extracellular matrix components [3]. This transition is highly relevant in the context of cardiovascular diseases, as it can lead to the impairment of endothelial function, the promotion of vascular remodeling, and the development of fibrosis, all of which contribute to the progression of vascular pathologies [4].

Hyperglycemia and its associated metabolites, such as methylglyoxal (MGO), have been shown to induce endothelial dysfunction and promote EndMT. MGO, a highly reactive di-carbonyl compound, has been shown to result in a state of glycation stress in endothelial cells, leading to elevated levels of advanced glycation end products and the activation of multiple cellular stress pathways [5]. MGO-induced endothelial cell stress has been linked to the induction of oxidative stress, inflammatory responses, and the activation of apoptotic signaling cascades. These cellular insults can ultimately result in endothelial dysfunction, impaired angiogenesis, and vascular complications [6].

Multiple mechanisms are involved in maintaining endothelial homeostasis. Sesterin2 (SESN2) has emerged as a critical player in this regard due to its ability to influence various cellular processes, owing to its multifaceted functions [7]. SESN2, as a cytoprotective protein, has been shown to exert protective effects against oxidative stress, inflammation, and other pathological stimuli leading to endothelial dysfunction. SESN2 can regulate various signaling pathways, such as the AMP-activated protein kinase (AMPK) and the mammalian target rapamycin (mTOR) pathways. These signaling responses are crucial for maintaining proper endothelial function and preventing the promotion of EndMT [8]. Several studies have shed light on the protective role of SESN2 in maintaining endothelial homeostasis. For example, we have previously shown that SESN2 alleviates oxidative stress-induced endothelial dysfunction by upregulating antioxidant defense systems and promoting the activation of the AMPK pathway [9]. Moreover, SESN2 has been reported to reduce inflammation and protect endothelial cells against the detrimental effects of pro-inflammatory insults [10].

Despite the established protective role of SESN2 in endothelial cells, the impact of its suppression on EndMT and MGO-induced endothelial dysfunction remains to be elucidated. Previous studies have shown that diabetes is associated with a deficiency in SESN2 expression. In a study of diabetic nephropathy, SESN2 mRNA expression was significantly lower in diabetic patients compared to healthy controls. This reduction in SESN2 expression was negatively correlated with albuminuria, a key marker of diabetic kidney disease progression [11]. Similarly, kidney biopsies from diabetic patients showed a reduced SESN2 expression compared to the controls [12]. The mRNA expression of *SESN2* was significantly reduced in diabetic mice compared to the controls. The induction of SESN2 in these mice resulted in improved insulin sensitivity and reduced glucose levels [13]. Therefore, to emulate this situation experimentally in vitro, we have suppressed SESN2 expression to study the impact of Sesn2 deficiency in endothelial cells exposed to a diabetic injury (MGO) on EndMT. We hypothesize that the suppression of SESN2 promotes the process of EndMT and further exacerbates the detrimental effects of MGO on inducing EndMT, thereby contributing to the pathophysiological mechanisms underlying diabetic vascular complications. In this study, we aim to elucidate the relationship between SESN2 deficiency and the promotion of EndMT in the context of diabetes-induced endothelial dysfunction. Understanding these mechanisms may provide insights into potential therapeutic targets for preventing or reversing endothelial dysfunction in diabetic patients and preventing the progression of cardiovascular diseases.

## 2. Results

### 2.1. Effect of MGO Treatment on the Expression of SESN2 in Endothelial Cells

**Figure 1:**
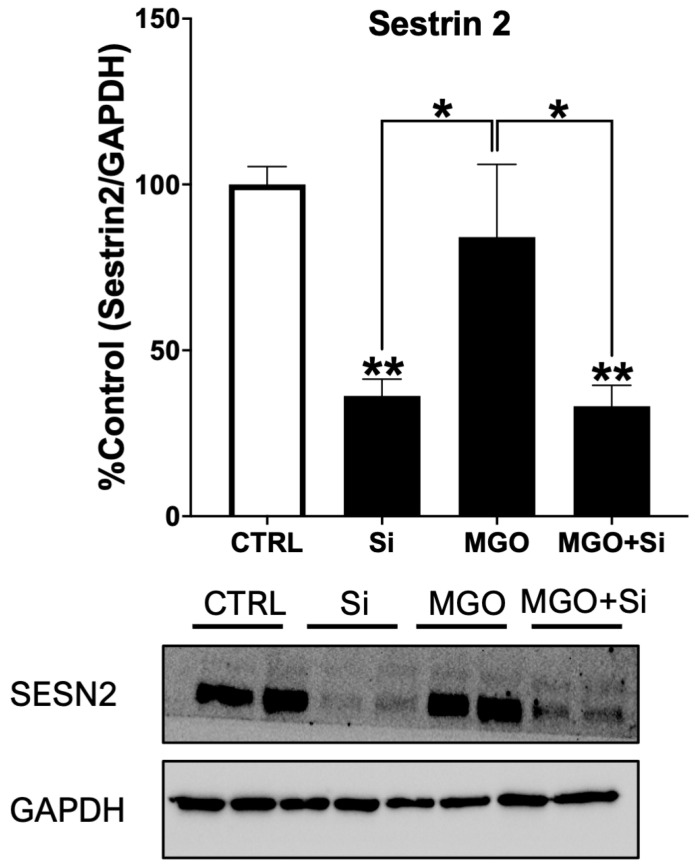
Effect of MGO treatment on the expression of SESN2 in endothelial cells. Western bot analysis and densitometry data of SESN2 protein expression normalized against loading control GAPDH and expressed as a percentage (%) of the untreated group (CTRL) (n = 4 in each group). Cells were left untreated or incubated with SESN2 siRNA duplexes for 48 h and then either exposed or not exposed to MGO (600 μM for 18 h). Data are presented as mean ± S.E.M. * *p* < 0.05, ** *p* < 0.01 vs. CTRL or indicated groups.

SESN2 is a stress-inducible cytoprotective protein whose expression increases in response to different types of cellular stress [8]. We investigated the impact of MGO on the expression of SESN2 in EA.hy926 endothelial cells proficient or deficient for *SESN2*. As anticipated, transfection of cells with *SESN2* siRNA duplexes resulted in a marked decrease in SESN2 protein expression compared to untreated cells (Figure 1). Furthermore, exposure of cells to the stressor MGO dramatically enhanced SESN2 protein expression, an effect that was notably prevented in cells transfected with *SESN2* siRNA (Figure 1).

### 2.2. Effect of SESN2 Silencing on the mRNA Expression Levels of Endothelial and Mesenchymal Markers in Endothelial Cells Subjected to MGO

**Figure 2:**
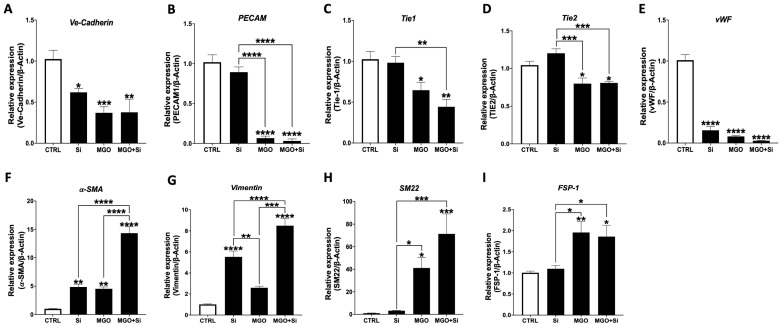
Effect of *SESN2* silencing on the mRNA expression levels of endothelial and mesenchymal markers in endothelial cells subjected to MGO. Relative mRNA expression levels of endothelial markers: *VE-Cadherin* (**A**), *PECAM* (**B**), *TIE1* (**C**), *TIE2* (**D**), *vWF* (**E**), and mesenchymal markers *α-SMA* (**F**), *Vimentin* (**G**), *SM22* (**H**) and *FSP-1* (**I**) normalized against housekeeping gene *β-actin* (n = 6 in each group). Cells were left untreated or incubated with *SESN2* siRNA duplexes for 48 h, and then exposed or not to MGO (600 μM for 18 h). Data are presented as mean ± S.E.M. * *p* < 0.05, ** *p* < 0.01, *** *p* < 0.001, **** *p* < 0.0001, vs. CTRL or indicated groups.

EndMT is emerging as a key player in CVD, including atherosclerosis. This process entails a sequence of cellular modifications, encompassing alterations in gene and protein expression, characterized by the loss of endothelial markers such as VE-Cadherin and the acquisition of mesenchymal markers like α-smooth muscle actin (α-SMA). We examined the impact of *SESN2* silencing on EndMT both in the presence and absence of MGO. Silencing *SESN2* alone markedly diminished the mRNA expression of endothelial markers, *VE-cadherin*, and the von Willebrand factor (*vWF*), with a tendency towards the reduced expression of *PECAM*, *Tie1*, and *Tie2* (Figure 2A–E). In contrast, the silencing of *SESN2* markedly elevated the expression of mesenchymal markers *α-SMA* and *vimentin*, alongside a tendency for increasing levels of *SM22* and *FSP-1* (Figure 2F–I). The MGO treatment alone resulted in a substantial decrease in all endothelial markers, while simultaneously dramatically increasing mesenchymal markers. Significantly, the silencing of *SESN2* intensified the impact of MGO, leading to a marked downregulation of endothelial markers and a substantial overexpression of mesenchymal markers (Figure 2A–I).

### 2.3. Effect of SESN2 Silencing on the Protein Expression of Endothelial and Mesenchymal Markers in Endothelial Cells Subjected to MGO

**Figure 3:**
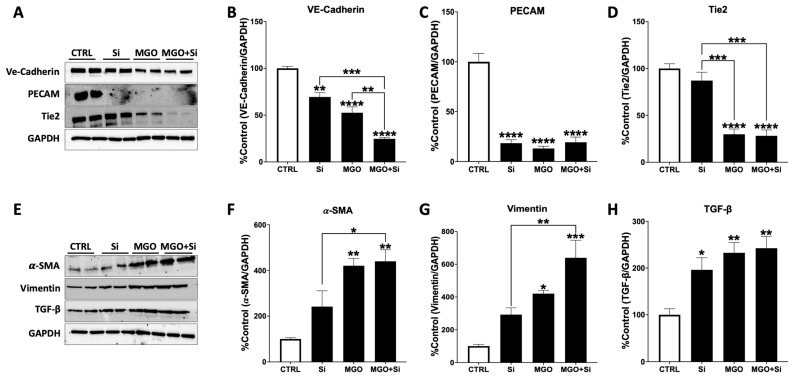
Effect of *SESN2* silencing on the protein expression of endothelial and mesenchymal markers in endothelial cells subjected to MGO. (**A**) Western bot analysis of endothelial markers: VE-Cadherin, PECAM, and Tie2. (**B**–**D**) Densitometry data of protein expression of VE-Cadherin (**B**), PECAM (**C**), and Tie2 (**D**) normalized against loading control GAPDH and expressed as a percentage (%) of the untreated group (CTRL). (**E**) Western bot analysis of mesenchymal markers: α-SMA, Vimentin, and TGF-β. (**F**–**H**) Densitometry data of protein expression of α-SMA (**F**), Vimentin (**G**), and TGF-β (**H**) normalized against loading control GAPDH and expressed as a percentage (%) of the untreated group (CTRL). Cells were left untreated or incubated with *SESN2* siRNA duplexes for 48 h, and then exposed to MGO (600 μM for 18 h). Data are presented as mean ± S.E.M (n = 4 in each group). * *p* < 0.05, ** *p* < 0.01, *** *p* < 0.001, **** *p* < 0.0001, vs. CTRL or indicated groups.

To further validate the effects of *SESN2* silencing on EndMT, we analyzed the protein expression of endothelial and mesenchymal markers in cells subjected to MGO treatment and those that were not (Figure 3). Silencing *SESN2* alone markedly diminished the protein levels of VE-Cadherin and PECAM, with a tendency towards reduced Tie2 expression. MGO treatment markedly reduced the expression of VE-Cadherin, PECAM, and Tie2. Conversely, mesenchymal markers exhibited a tendency for higher expression of α-SMA and vimentin in *SESN2*-silenced cells, accompanied by a notable elevation in TGF-β protein levels. The MGO treatment alone markedly increased the expression of α-SMA, vimentin, and TGF-β proteins. In *SESN2*-deficient endothelial cells, MGO administration markedly intensified EndMT by diminishing endothelial markers (VE-Cadherin, PECAM, and Tie2) and promoting mesenchymal markers (α-SMA, vimentin, and TGF-β) (Figure 3).

### 2.4. Effect of SESN2 Silencing on the Expression of Oxidative Stress Markers in Endothelial Cells Subjected to MGO

**Figure 4:**
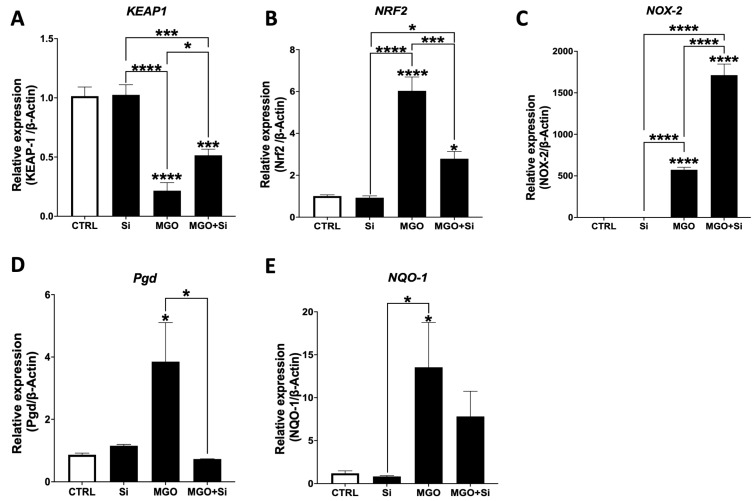
Effect of *SESN2* silencing on the expression of oxidative stress markers in endothelial cells subjected to MGO. Relative mRNA expression levels of *KEAP-1* (**A**), *NRF-2* (**B**), *NOX-2* (**C**), *PGD* (**D**), and *NQO-1* (**E**) normalized against housekeeping gene *β-actin* (n = 6 in each group). Cells were left untreated or incubated with *SESN2* siRNA duplexes for 48 h, and then exposed to MGO (600 μM for 18 h). Data are presented as mean ± S.E.M. * *p* < 0.05, *** *p* < 0.001, **** *p* < 0.0001, vs. CTRL or indicated groups.

Considering that *SESN2* silencing exacerbated MGO-induced EndMT, we subsequently examined the impact of MGO on oxidative stress markers in *SESN2*-deficient cells. SESN2 facilitates its antioxidant actions, in part, by degrading Keap-1, which subsequently activates Nrf-2. In cells exhibiting normal SESN2 expression (not silenced) treated with MGO, we detected a substantial decrease in *Keap-1* (Figure 4A) and a notable increase in *Nrf-2* mRNA expression (Figure 4B). MGO treatment markedly increased the mRNA expression of *Nox-2*, *Pgd*, and *NQO-1*. In *SESN2*-deficient cells exposed to MGO, there was a notable elevation in *Keap-1* expression, accompanied by a substantial decrease in *Nrf-2* and *Pgd* mRNA levels, compared to the MGO-treated group.

### 2.5. Effect of SESN2 Silencing on the Expression of Inflammatory Markers in Endothelial Cells Subjected to MGO

**Figure 5:**
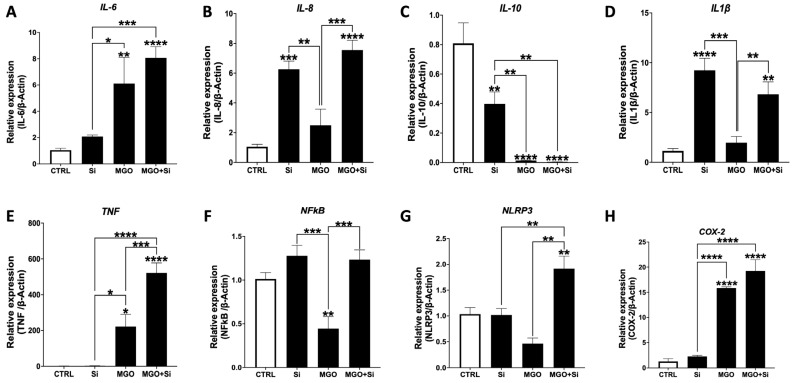
Effect of SESN2 silencing on the expression of inflammatory markers in endothelial cells subjected to MGO. Relative mRNA expression levels of *IL-6* (**A**), *IL-8* (**B**), *IL-10* (**C**), *IL-1β* (**D**), *TNF-α* (**E**), *NF-κB* (**F**), *NLRP3* (**G**), and *COX-2* (**H**) normalized against housekeeping gene *β-actin* (n = 6 in each group). Cells were left untreated or incubated with *SESN2* siRNA duplexes for 48 h, and then exposed or not to MGO (600 μM for 18 h). Data are presented as mean ± S.E.M. * *p* < 0.05, ** *p* < 0.01, *** *p* < 0.001, **** *p* < 0.0001, vs. CTRL or indicated groups.

Inflammation is a primary driver of EndMT, with inflammatory cytokines serving a pivotal function in initiating and promoting the process [14]. We assessed the expression of many cytokines in *SESN2*-silenced cells, exposed or not exposed to MGO (Figure 5). The co-treatment of MGO and *SESN2* silencing resulted in a marked elevation in *IL-6* and *COX-2* mRNA levels in comparison to *SESN2* silencing alone. Moreover, the concurrent silencing of *SESN2* and MGO treatment markedly increased the mRNA expression levels of *IL-8*, *IL-1β*, *TNF-α*, *NF-κB*, and *NLRP3* in comparison to MGO treatment alone. Conversely, the silencing of *SESN2* alone resulted in a marked decrease in the gene expression of the anti-inflammatory cytokine *IL-10* compared to the control (Figure 5C). A more pronounced reduction in *IL-10* mRNA levels was noted in the *SESN2*-silenced cells exposed to MGO compared to *SESN2* silencing alone.

### 2.6. Impact of SESN2 Silencing on the Expression Levels of Adhesion Molecules in Endothelial Cells Subjected to MGO

**Figure 6:**
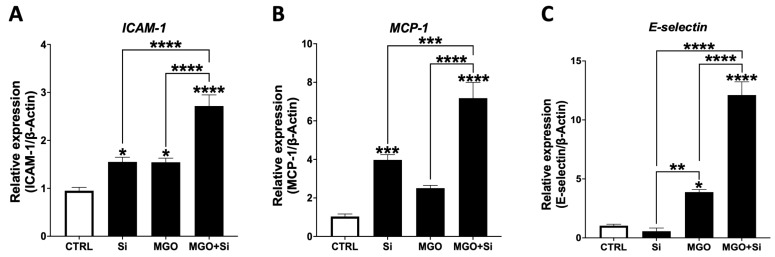
Impact of SESN2 silencing on the expression levels of adhesion molecules in endothelial cells subjected to MGO. Relative mRNA expression levels of *ICAM-1* (**A**), *MCP-1* (**B**), and *E-selectin* (**C**) normalized against housekeeping gene *β-actin* (n = 6 in each group). Cells were left untreated or incubated with *SESN2* siRNA duplexes for 48 h, and then exposed to MGO (600 μM for 18 h). Data are presented as mean ± S.E.M. * *p* < 0.05, ** *p* < 0.01, *** *p* < 0.001, **** *p* < 0.0001, vs. CTRL or indicated groups.

Adhesion molecules are essential in the EndMT process by facilitating the loss of endothelial cell-cell adhesion and fostering the development of mesenchymal features, including enhanced cell motility and invasion [15]. This process is governed by signaling pathways such as TGF-β. We assessed the gene expression levels of adhesion molecules *ICAM-1*, *MCP-1*, and *E-Selectin* in *SESN2*-silenced cells exposed or not to MGO (Figure 6). We observed a trend toward increased expression of *ICAM-1* and *MCP-1*, while a significant increase in *E-Selectin* mRNA expression was noted in the MGO-treated group compared to untreated cells. Notably, the combination of *SESN2* silencing with MGO treatment resulted in a substantial elevation of *ICAM-1*, *MCP-1*, and *E-Selectin* mRNA expression, surpassing the levels observed in both the *SESN2*-silenced and MGO-treated groups alone.

### 2.7. Impact of SESN2 Silencing on the Activation of EndMT Signaling Pathway in Endothelial Cells Exposed to MGO

**Figure 7:**
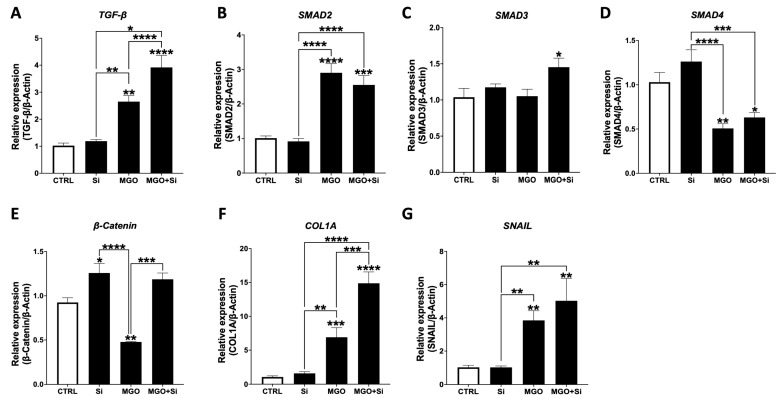
Impact of SESN2 silencing on the expression levels of TGF-β signaling and transcription factors in endothelial cells subjected to MGO. Relative mRNA expression levels of *TGF-β* (**A**), *SMAD2* (**B**), *SMAD3* (**C**), *SMAD4* (**D**), *β-Catenin* (**E**), *COL1A1* (**F**), and *SNAI1* (**G**) normalized against housekeeping gene *β-actin* (n = 6 in each group). Cells were left untreated or incubated with *SESN2* siRNA duplexes for 48 h, and then exposed or not exposed to MGO (600 μM for 18 h). Data are presented as mean ± S.E.M. * *p* < 0.05, ** *p* < 0.01, *** *p* < 0.001, **** *p* < 0.0001, vs. CTRL or indicated groups.

TGF-β signaling plays a central role in EndMT and is considered a primary driver of the process. Wnt/β-catenin signaling may also contribute to EndMT [16]. Moreover, the expression of transcription factors such as Snail, Slug, and Twist are induced in EndMT, which represses the expression of endothelial genes and upregulates the expression of mesenchymal genes [17]. We assessed the expression of TGF-β signal, β-catenin, and Snail as a transcription factor below (Figure 7).

TGF-β and SMAD2 mRNA expression were significantly induced upon MGO treatment. This induction was significantly higher in the group silenced for SESN2 and treated with MGO compared to the silenced group alone. The treated and silenced group had a significantly higher level of *SMAD3* mRNA expression compared to control. *SMAD4* expression levels were significantly lower in the MGO group as well as the MGO plus silencing group compared to the silencing group alone. Looking further at the *β-catenin* gene expression, silencing resulted in a significant rise in *β-catenin* mRNA levels compared to control. Silencing for *SESN2* followed by MGO treatment significantly increased *β-catenin* levels compared to the treated group. For both *COL1A1* and *SNAI1*, mRNA expression levels were significantly induced in the MGO and MGO and the silencing groups compared to both the control and silenced groups.

TGF-β signaling is a main regulator of EndMT and is regarded as the fundamental driver for this process, with Wnt/β-catenin signaling potentially playing a contributing role [17]. The expression of transcription factors, including Snail, Slug, and Twist, is elevated during EndMT, leading to the suppression of endothelial gene expression and the promotion of mesenchymal gene expression, therefore examining the expression of TGF-β signaling components, as well as β-catenin and Snail, which acts as a transcription factor (Figure 7). MGO treatment markedly enhanced the mRNA expression of *TGF-β* and *SMAD2*. The induction was much greater in the *SESN2*-silenced cells exposed to MGO compared to the silenced cells. *SMAD3* mRNA levels were markedly increased in the MGO-treated and *SESN2*-silenced cells compared to the control.

Conversely, *SMAD4* expression was diminished significantly in both the MGO-treated cells and *SENS2*-silenced cells exposed to MGO compared to the silenced group alone. Silencing *SESN2* resulted in a notable elevation of *β-catenin* mRNA levels compared to the control. The silencing of *SESN2* in conjunction with MGO treatment led to a notable enhancement in *β-catenin* expression compared to the MGO-only treated group. Finally, mRNA levels of both *COL1A1* and *SNAI1* were markedly elevated in the MGO-treated cells and SESN2-silenced cells exposed to MGO compared to untreated cells and the *SENS2*-silenced group.

## 3. Discussion

This study investigated the role of SESN2 in the EndMT induced by MGO, which is a well-known contributor to diabetic-related vascular complications. The downregulation of SESN2 expression using *SESN2* siRNA duplexes in human endothelial cells enhanced the detrimental effects of MGO. *SESN2* knockdown heightened the MGO-induced oxidative stress, as measured by reduced gene expression of antioxidant markers. Further, *SESN2* silencing enhanced the inflammatory response to MGO, as measured by increased levels of pro-inflammatory cytokines. A key finding of our study is that the absence of SESN2 has promoted EndMT, a process associated with endothelial dysfunction and vascular remodeling. This was shown by increased expression of mesenchymal markers and decreased expression of endothelial markers in *SESN2*-silenced cells. These results strongly suggest that SESN2 plays a critical protective role in endothelial cells against MGO-induced damage, and its downregulation may contribute to the pathogenesis of diabetic vascular complications.

Our study investigated the role of SESN2 in the context of diabetes. SESN2 is a critical player in managing cellular stress, and its levels can change depending on various factors, such as the stage of diabetes, the presence of other health conditions, and which tissues are being studied. The existing literature on SESN2 in diabetes shows mixed results. Some studies show that SESN2 levels are higher in diabetes, while others report a decline. This variability likely reflects the dynamic nature of SESN2’s role, which changes over time as the disease progresses. In the early stages of diabetes, SESN2 levels may rise as part of a protective response to oxidative stress, particularly in tissues like the endothelium, where it helps maintain normal vascular function. For instance, newly diagnosed diabetics show higher SESN2 levels compared to controls [18], which may reflect the body’s initial attempts to combat the negative effects of elevated blood sugar. However, as the disease advances, this protective mechanism can become overwhelmed, and SESN2 levels can decrease, potentially due to chronic stress and the development of complications.

A study by Mao et al. showed that serum SESN2 levels of all diabetic patients were significantly higher than healthy controls. Interestingly, within the diabetic populations, SESN2 levels may vary based on complications; diabetic patients who developed diabetic peripheral neuropathy had notably lower serum SESN2 levels compared to diabetic patients without diabetic peripheral neuropathy [19]. Similarly, serum SESN2 levels in diabetic patients with coronary heart diseases were significantly lower compared with the diabetic group without complications [20]. A recent study from our group further supports this idea [21]. Abdelsalam et al. reported that circulating levels of SESN2 were significantly lower in the diabetic group compared to healthy controls [21]. This suggests that as diabetes progresses, SESN2 may no longer be able to keep up with the oxidative damage and inflammation associated with the disease. Our current findings add weight to the theory that SESN2 levels are reduced as the disease advances, especially in the context of risk factors associated with cardiovascular disease. These findings underscore the importance of understanding how SESN2 regulation evolves over time in diabetes. SESN2 seems to play a dual role—protective in the early stages of the disease but potentially contributing to metabolic dysfunction as the disease becomes more chronic. This is the rationale for our current study, which investigates the role of SESN2 in MGO-induced EndMT in endothelial cells. Understanding how SESN2 influences this process could provide valuable insights into the pathogenesis of diabetic-induced vascular complications.

The most significant finding of our study is the promotion of EndMT in *SESN2*-silenced cells exposed to MGO. This observation aligns with the work of Hwang et al. [22], who showed that *SESN2* knockdown increases lipopolysaccharide (LPS)-induced oxidative stress, apoptosis, and fibrotic reactions in heart tissues. While their research model was cardiomyocytes, it provided supporting evidence for SESN2’s role in regulating processes involved in EndMT, such as oxidative stress and fibrosis. The increased expression of mesenchymal markers and decreased expression of endothelial markers in *SESN2*-deficient cells suggest that SESN2 is a key regulator of endothelial cell identity under metabolic stress. Moreover, the induction of EndMT was accompanied by an upregulation in the TGF-β signaling pathway and SNAI1 transcription factor. This finding is in agreement with Ma et al. [23], who highlighted that EndMT is induced by TGF-β which strongly upregulates the transcription factor SNAI1 [23]. Our results suggest that SESN2 may play a role in maintaining this balance, potentially by modulating the expression or activity of these key transcription factors.

The decreased expression of antioxidant enzymes observed in *SESN2*-silenced cells exposed to MGO aligns with the established antioxidant functions of SESN2. SESN2 has been previously shown to possess antioxidant properties through its regulation of the Nrf2 pathway and autophagy, which helps maintain cellular redox balance. We have previously shown that *SESN2* silencing exacerbated oxidative stress in endothelial cells challenged with an endoplasmic reticulum (ER) stress pharmacological inducer [9]. Our data support this notion, highlighting the importance of SESN2 in controlling oxidative stress in endothelial cells. In diabetes, where MGO levels are elevated, oxidative stress is a key contributor to endothelial dysfunction, and the lack of SESN2 exacerbates this process. This observation raises the possibility that strategies aimed at enhancing SESN2 expression or function could help attenuate oxidative damage in endothelial cells and thus ameliorate the vascular complications observed in diabetes. This finding is also consistent with the work of Hwang et al. [24], who demonstrated that *SESN2* knockdown increases ER stress, ROS production, and cell death in endothelial cells [24]. The amplified oxidative stress in *SESN2*-deficient cells underscores its importance in maintaining redox balance, a critical factor in preventing endothelial dysfunction in diabetic conditions.

Furthermore, we observed that *SESN2* knockdown enhanced the inflammatory response to MGO, as evidenced by the increased levels of pro-inflammatory cytokines. Inflammation plays a central role in developing vascular complications in diabetes, and endothelial cells are key mediators of this process [25]. The elevation in cytokine levels following *SESN2* silencing suggests that SESN2 has an anti-inflammatory role in endothelial cells, which is consistent with previous reports indicating its involvement in regulating inflammatory responses in other cell types [26]. The activation and secretion of IL-1β and IL-18 are mediated by NLRP3 inflammasomes. NLRP-3 inflammasomes are important mediators of inflammation. They are molecular complexes that recruit and transform caspase-1 and caspase-11 into their active forms, which leads to cleavage and activation of IL-1β and IL-18 and promotion of inflammatory cell recruitment [27]. Various stress signals, such as reactive oxygen species (ROS) and AGEs, activate NLRP-3 inflammasomes. The activation of NLRP-3 inflammasome was detected in endothelial cells and podocytes of diabetic humans, which aggravated diabetic nephropathy [28]. Our results also showed a significant increase in the gene expression of *IL-1β* and *NLRP3* in *SESN2*-deficient cells exposed to MGO. These observations were in line with those reported by Wu et al. [29], where *SESN2*-deficient mice had a significantly increased expression of NLRP3 and IL-1β upon LPS exposure [29]. In the context of diabetes, chronic inflammation in the endothelium contributes to the development of atherosclerosis and other vascular pathologies [30]. Therefore, our results imply that the loss of SESN2 may not only enhance oxidative stress but also exacerbate the inflammatory milieu within the vasculature, further promoting the progression of diabetic vascular complications.

This study, although offering significant insights into the function of SESN2 in endothelial dysfunction and EndMT, exhibits some limitations. It primarily utilizes an in vitro model employing EA.hy926 endothelial cells, which may not completely mimic the intricacies of the in vivo endothelium environment. The acute exposure to MGO (600 μM for 18 h) employed to replicate diabetic conditions may not fully represent the chronic nature of hyperglycemia in diabetes. The siRNA-mediated knockdown of SESN2 is successful but does not entirely abolish SESN2 expression; a complete knockout model could yield more conclusive results. The investigation of molecular pathways is constrained, and the lack of rescue trials and in vivo validation somewhat diminishes the direct therapeutic applicability of the results. Overcoming these limitations in subsequent studies will be essential for enhancing our comprehension of SESN2’s involvement in endothelial activity and its viability as a therapeutic target in diabetic vascular complications.

Our study contributes to the growing body of evidence linking SESN2 to cellular stress responses in the context of metabolic disorders. While the focus of the literature was on the role of SESN2 as a protective protein against oxidative stress and cellular damage, our work sheds light on the specific importance of SESN2 in endothelial cells exposed to metabolic stressors such as MGO. SESN2’s protective effects may be not only limited to its activity as an antioxidant but also involve playing a role in modulating critical signaling pathways that govern cell survival, inflammation, and vascular homeostasis. Further investigations into the molecular mechanisms by which SESN2 regulates these pathways in endothelial cells are needed to fully elucidate its protective role in diabetic cardiovascular complications.

## 4. Materials and Methods

### 4.1. Cell Culture

EA.hy926 (ATCC^®^ CRL-2922™, ATCC, Manassas, VA, USA) endothelial cells were cultured in high glucose Dulbecco’s Modified Eagle’s Medium (DMEM) supplemented with 10% fetal bovine serum (FBS), 1% penicillin/streptomycin (P/S), sodium pyruvate (1%), and L-glutamine (1%) from Gibco (Thermo Scientific, Waltham, MA, USA). Cells were left to adhere and proliferate at 37 °C, 5% carbon dioxide (CO_2_), and 95% humidity within an incubator. Cells were used in experimental studies up to passage 15.

### 4.2. Cell Treatments

All treatments were conducted in 6-well plates, with each well seeded with 200,000 endothelial cells. Following a 24-h adhesion period, cells were treated with methylglyoxal (MGO; 600 μM, 18 h; Sigma-Aldrich, Darmstadt, Germany), a precursor of advanced glycation end-products (AGEs), to pharmacologically simulate diabetic conditions.

*SESN2* and control siRNA duplexes were designed and synthesized by Integrated DNA Technologies (IDT, Coralville, IA, USA) for *SESN2* gene silencing assays. Briefly, 50,000 endothelial cells were seeded onto 6-well plates and allowed to adhere for 24 h. Then, cells were transfected with either control or *SESN2* siRNA duplexes following the manufacturer’s procedure. For transfection preparation, 250 μL of OptiMEM solution (Thermo Scientific, Waltham, MA, USA) was dispensed into two distinct Falcon tubes. INTERFERin^®^ transfection reagent (Polyplus, Illkirch, France) was solubilized in one tube, whereas siRNA duplexes were solubilized in another. The contents of the two tubes were thereafter combined, properly mixed, and incubated at room temperature for 20 min. After incubation, 500 μL of the transfection mixture was introduced into each well containing 2 mL of serum- and antibiotic-free medium. Eight hours later, the medium was substituted with a fresh medium containing 10% FBS but without antibiotics. Cells were then cultured for 48 h following transfection before undergoing treatment with or without the pharmacological agent MGO (600 μM, 18 h; Sigma-Aldrich).

### 4.3. Western Blot Analysis

Following treatments, culture plates were placed on ice. The medium was meticulously aspirated and removed, and the cells were washed twice with ice-cold phosphate-buffered saline (PBS; Thermo Scientific, Waltham, MA, USA) to eliminate any remaining medium. For cell lysis, cold radioimmunoprecipitation assay (RIPA) buffer, including Tris (0.5 M, pH 6.8) and sodium dodecyl sulfate (SDS, 20%; Thermo Scientific), was supplemented with a protease and phosphatase inhibitor cocktail tablet (Thermo Scientific). The resultant protein lysates were gathered for subsequent analysis. Protein concentrations were determined utilizing the bicinchoninic acid (BCA) assay (Thermo Scientific). Equal quantities of protein (10–20 µg) were separated using SDS-PAGE gels (8–12%, depending on the molecular weight of the target proteins) and subsequently transferred to nitrocellulose membranes (Thermo Scientific, Waltham, MA, USA). Membranes were incubated for one hour at room temperature in tris-buffered saline with 0.1% Tween 20 (TBS-T; Sigma-Aldrich, Hamburg, Germany) containing 5% bovine serum albumin (BSA). Following blocking, membranes were washed with TBS-T and incubated overnight at 4 °C on a shaker with the following primary antibodies: SESN2 (#8487S), vascular endothelial (VE)-cadherin (#2500S), platelet endothelial cell adhesion molecule (PECAM-1) (#77699S), tyrosine kinase with immunoglobin and EGF homology domains 2 (Tie2) (#7403S), vimentin (#5741S), transforming growth factor (TGF)-β (#3711S), GAPDH (#2118S) (Cell Signalling Technology, Danvers, MA, USA), and alpha-smooth muscle actin (α-SMA) (#ab7817) (Abcam, Cambridge, UK). The next day, membranes were washed with TBS-T and incubated for one hour at ambient temperature with horseradish peroxidase (HRP)-conjugated secondary antibodies (anti-rabbit #7074S or anti-mouse #7076S; Cell Signalling Technology) at a dilution of 1:10,000. Protein bands were visualized with Optiblot ECL reagent (Abcam, Cambridge, UK) and captured using the Bio-Rad ChemiDoc MP imaging system (Bio-Rad, Hercules, CA, USA). Densitometric analysis of protein bands was conducted using Image Lab Software for Mac version 6.1 (Bio-Rad, Hercules, CA, USA).

### 4.4. Total RNA Isolation and Gene Expression Analysis

Following treatments, cells were washed, trypsinized, and harvested. Total RNA was extracted from cell pellets using the innuPREP RNA Mini Kit (Analytikjena, Berlin, Germany) according to the manufacturer’s instructions. The purity and concentration of RNA were evaluated using the NanoDrop 2000 spectrophotometer (Thermo Scientific, Waltham, MA, USA). Complementary DNA (cDNA) was synthesized from 1000 ng of RNA via reverse transcription using the RevertAid Reverse Transcription Kit (Thermo Scientific) and an oligo(dT)12-18 primer, following the kit’s protocol. Subsequently, 2 ng of synthesized cDNA was amplified utilizing Luna^®^ Universal qPCR Master Mix (New England Biolabs, Ipswich, MA, USA) and human-specific primers for target genes on an Applied Biosystems QuantStudio™ 5 Real-Time PCR System (Thermo Scientific, Waltham, MA, USA). The experiment was conducted using six independent biological replicates by using distinct passages of EA.hy926 cells to guarantee reproducibility or results. Relative gene expression was assessed by the comparative ΔΔCt method, with mRNA levels normalized to *β-actin* as the housekeeping reference gene. Results were presented as fold changes compared to the control group. Primer sequences were obtained from the Primer Bank and synthesized by Sigma-Aldrich (Hamburg, Germany) and Integrated DNA Technologies (Coralville, IA, USA). The primer sequences used in the investigation are detailed in Table 1.

### 4.5. Statistical Analysis

Results are expressed as mean ± SEM, and n represents the number of biological repeats. GraphPad Prism^®^ 10.4.0 software for Mac was used to perform the statistical analyses. The normality of data was tested each time using the Shapiro–Wilk normality test. For data with a Gaussian distribution, a one-way ANOVA followed by Tukey’s multiple comparisons post hoc test was applied for group comparison. For non-Gaussian data, the Kruskal–Wallis test, followed by Dunn’s multiple comparisons post hoc test, was used for group comparison. Statistical significance was defined as a two-tailed *p* ≤ 0.05.

## 5. Conclusions

Our study underscores the critical role of SESN2 in protecting endothelial cells from MGO-induced damage, highlighting its importance in maintaining endothelial function and preventing EndMT. The downregulation of SESN2 enhances oxidative stress, inflammation, and EndMT, processes that are central to the pathogenesis of diabetic vascular complications. These findings suggest that strategies aimed at modulating SESN2 expression or activity could offer a promising therapeutic approach for mitigating the vascular complications associated with diabetes. Future studies should focus on understanding the precise molecular mechanisms through which SESN2 exerts its protective effects and evaluate potential therapeutic interventions targeting SESN2 in diabetic vascular diseases.

## Figures and Tables

**Table 1:** List of human primers used in the study.

Target Genes	Forward	Reverse
*ACTB*	AGCCTCGCCTTTGCCGAT	CTGACCCATGCCCACCATCA
*CDH5*	TTGGAACCAGATGCACATTGAT	TCTTGCGACTCACGCTTGAC
*PECAM-1*	AACAGTGTTGACATGAAGAGCC	TGTAAAACAGCACGTCATCCTT
*TIE1*	AAGCAGACAGACGTGATCTGG	GCACGATGAGCCGAAAGAAG
*TIE2*	TCCGCTGGAAGTTACTCAAGA	GAACTCGCCCTTCACAGAAATAA
*vWF*	CCGATGCAGCCTTTTCGGA	TCCCCAAGATACACGGAGAGG
*ACTA2*	AAAAGACAGCTACGTGGGTGA	GCCATGTTCTATCGGGTACTTC
*VIM*	AGTCCACTGAGTACCGGAGAC	CATTTCACGCATCTGGCGTTC
*TAGLN*	AGTGCAGTCCAAAATCGAGAAG	CTTGCTCAGAATCACGCCAT
*AIM1*	GCAGCAACCTAGTGTACTTCTT	CTGGTGTCAGCCCTAACCC
*Keap-1*	GGCTGTCCTCAATCGTCTCC	TCTGTTTCCACATCGTAGCG
*NOX-2*	ACCGGGTTTATGATATTCCACCT	GATTTCGACAGACTGGCAAGA
*NQO-1*	AGCCCAGATATTGTGGCCG	CCTTTCAGAATGGCTGGCAC
*Pgd*	GTTCCAAGACACGATGGCAAAC	CACCGAGCAAAGACAGCTTCTC
*IL-6*	AAATTCGGTACATCCTCGACGG	GGAAGGTTCAGGTTGTTTTCTGC
*IL-8*	ACTGAGAGTGATTGAGAGTGGAC	AACCCTCTGCACCCAGTTTTC
*IL-10*	ACTTTAAGGGTTACCTGGGTTGC	TCACATGCGCCTTGATGTCTG
*IL1B*	ATGATGGCTTATTACAGTGGCAA	GTCGGAGATTCGTAGCTGGA
*TNF*	GAGGCCAAGCCCTGGTATG	CGGGCCGATTGATCTCAGC
*NKB1*	AACAGAGAGGATTTCGTTTCCG	TTTGACCTGAGGGTAAGACTTCT
*NLRP*	CGTGAGTCCCATTAAGATGGAGT	CCCGACAGTGGATATAGAACAGA
*COX-2*	CTGGCGCTCAGCCATACAG	CGCACTTATACTGGTCAAATCCC
*ICAM1*	ATGCCCAGACATCTGTGTCC	GGGGTCTCTATGCCCAACAA
*MCP-1*	CAGCCAGATGCAATCAATGCC	TGGAATCCTGAACCCACTTCT
*SELE*	AGAGTGGAGCCTGGTCTTACA	CCTTTGCTGACAATAAGCACTGG
*TGB1*	CAATTCCTGGCGATACCTCAG	GCACAACTCCGGTGACATCAA
*SMAD2*	CGTCCATCTTGCCATTCACG	CTCAAGCTCATCTAATCGTCCTG
*SMAD*	CCATCTCCTACTACGAGCTGAA	CACTGCTGCATTCCTGTTGAC
*SMAD4*	CTCATGTGATCTATGCCCGTC	AGGTGATACAACTCGTTCGTAGT
*CTNNB1*	AGCTTCCAGACACGCTATCAT	CGGTACAACGAGCTGTTTCTAC
*COL1A1*	GTGCGATGACGTGATCTGTGA	CGGTGGTTTCTTGGTCGGT
*SNAI1*	TCGGAAGCCTAACTACAGCGA	AGATGAGCATTGGCAGCGAG

## Data Availability

All relevant data are included within the manuscript. Additional data or information can be provided by the corresponding author upon reasonable request.
